# Preliminary Usability Assessment of a Rule-Based Digital Self-Monitoring Platform for Patients With Brain Tumors Toward Digital Early Warning Systems: Pilot Feasibility Study

**DOI:** 10.2196/87928

**Published:** 2026-04-17

**Authors:** Yourack Lee, HyeongGuk SON, Joonho Byun

**Affiliations:** 1 Biomedical Research Center Korea University Guro Hospital Seoul, Guro-gu Republic of Korea; 2 Medical Device Usability Test Center Korea University Guro Hospital Seoul, Guro-gu Republic of Korea; 3 Department of Neurosurgery Korea University College of Medicine Korea University Guro Hospital Seoul, Guro-gu Republic of Korea

**Keywords:** brain tumor, neurosurgery, mobile health, self-monitoring, early warning systems

## Abstract

**Background:**

Postoperative follow-up after brain tumor surgery is typically limited to intermittent clinic visits, leaving subtle neurological or general deterioration between visits underrecognized. Digital self-monitoring platforms may help fill this gap, but evidence in neuro-oncology is scarce, particularly regarding how patient-reported symptom trajectories can feed into future data-driven early warning systems.

**Objective:**

This study aimed to evaluate the feasibility, use patterns, and preliminary usability of a smartphone or web-based self-monitoring system for patients after brain tumor surgery and to explore simple rule-based digital alerts as a first step toward an advanced digital early warning framework.

**Methods:**

We conducted a single-center prospective pilot study including adults discharged after brain tumor surgery who had access to a smartphone and could use a web app. Participants completed brief symptom surveys consisting of 51 binary items across 7 symptom domains, with an automatically calculated daily total score and score history visualization. Feasibility was assessed by enrollment, retention, submission counts, and submission rates. A total of 4 interpretable alert rules based on current score, short-term worsening, new-onset symptom combinations, and persistence across domains were evaluated using each patient’s last 3 submissions as the analytic unit. Clinical deterioration was defined a priori as objective decline in performance status, new neurological deficit, radiologic progression, or clinically significant laboratory changes. Rule performance metrics and bootstrap CIs were computed. Usability and acceptability were evaluated using the System Usability Scale and additional adherence-related items.

**Results:**

Of 64 enrolled patients, 30 (47%) with ≥3 submissions formed the analysis cohort (median age 57, IQR 47.2–64.5 years; n=12.9, 43% malignant tumors); 6 (20%) experienced clinical deterioration during follow-up. Patients contributed a median of 8.5 submissions (mean 19.03, SD 30.12) at 1.7 surveys per week on average, indicating sustained but heterogeneous engagement. The best-performing rule, based on net short-term score increase, achieved an area under the receiver operating characteristic of 0.88, with sensitivity 0.83, specificity 0.92, and accuracy 0.90 on the last-window dataset, outperforming rules based solely on current score or multidomain persistence. Among 23 app users who completed the System Usability Scale, the mean score was 84.0, reflecting high perceived usability; higher-frequency users reported stronger perceived usefulness and habit-driven use.

**Conclusions:**

This pilot study demonstrates that a smartphone or web-based self-monitoring platform for patients with brain tumor is feasible and well accepted and that simple, transparent rules applied to longitudinal symptom scores show potential to capture early signals of clinical deterioration. However, given the small sample size, these predictive metrics are preliminary and require rigorous validation in larger, independent cohorts. These findings support further development of integrated digital early warning systems that combine patient-reported trajectories with clinical and physiological data to enhance postoperative neurosurgical care.

## Introduction

Advances in neurosurgical techniques have markedly improved survival and functional outcomes in patients with brain tumors [1,2]; however, postoperative management remains one of the most complex challenges in in the field of neurosurgery [3,4]. After neurological surgery, patients often experience fluctuating symptoms such as headache, fatigue, cognitive decline, or subtle sensorimotor changes that may precede significant clinical deterioration [2,5-7]. Despite these risks, most follow-up systems still rely on intermittent hospital visits and subjective recall, leaving a critical gap in the continuous monitoring of patients’ neurological and general well-being [8,9].

The expansion of digital health technologies, particularly smartphone apps and wearable devices such as smartwatches and smart rings, has created new possibilities for personalized and remote patient care [10-13]. Nevertheless, tools specifically designed for patient self-care remain scarce, and most existing digital monitoring systems are generalized, without the sensitivity required to detect subtle neurological changes. Patients with brain tumors constitute a rare and highly specialized group [14]. Because most specialists are concentrated in tertiary medical centers, and their number is limited, a considerable gap persists between apparent and actual accessibility to specialized care [15,16]. As a result, many patients struggle to receive timely evaluation and guidance for minor symptoms at primary or secondary health care institutions [17,18]. This imbalance often leads to 2 extremes: some patients with mild or nonspecific symptoms seek tertiary care unnecessarily out of anxiety or uncertainty, while others with neurological deficits delay seeking medical attention until substantial deterioration has occurred [8,19].

This study was designed to address these challenges by developing a self-report-based symptom monitoring system that incorporates simple rule-based alert algorithms. The system aims to support patients in managing mild symptoms independently, while providing early alerts for those showing signs of deterioration. To assess the practical feasibility of this approach, we designed and implemented a smartphone app to collect longitudinal patient-reported symptom data after brain tumor surgery. This pilot study evaluates the feasibility, usability, and preliminary applicability of the system as an initial step toward establishing a robust digital early warning framework for postoperative neurological risk detection. The findings from this work are expected to provide essential evidence for future large-scale integration of clinical, biological, and physiological data into a comprehensive digital ecosystem for continuous brain health monitoring.

## Methods

### Study Design and Setting

We conducted a single-center, prospective pilot cohort study to evaluate the feasibility and usability of a smartphone or web-based self-monitoring system for patients after brain tumor surgery during outpatient follow-up.

### Ethical Considerations

The study protocol was approved by the institutional review board of Korea University Guro Hospital (2025GR0177). Written informed consent was obtained from all participants prior to study enrollment. To ensure privacy and confidentiality, all collected data were rigorously deidentified, and patient-level information was stored on a secure, encrypted clinical research server accessible only to authorized personnel. Participants did not receive any financial compensation for their involvement in this study.

### Participants and Recruitment

Eligible participants were adults discharged after brain tumor surgery at our institution between April 2025 and October 2025. The participant eligibility criteria are presented in Textbox 1.

**Participant eligibility criteria.**

**Inclusion criteria**

- Aged ≥19 years
- Consent to use the study app
- Access to a smartphone or PC
- Korean language proficiency

**Exclusion criteria**

- Severe cognitive impairment
- Life expectancy <6 months
- Investigator judgment of inability to participate

**Figure 1:**
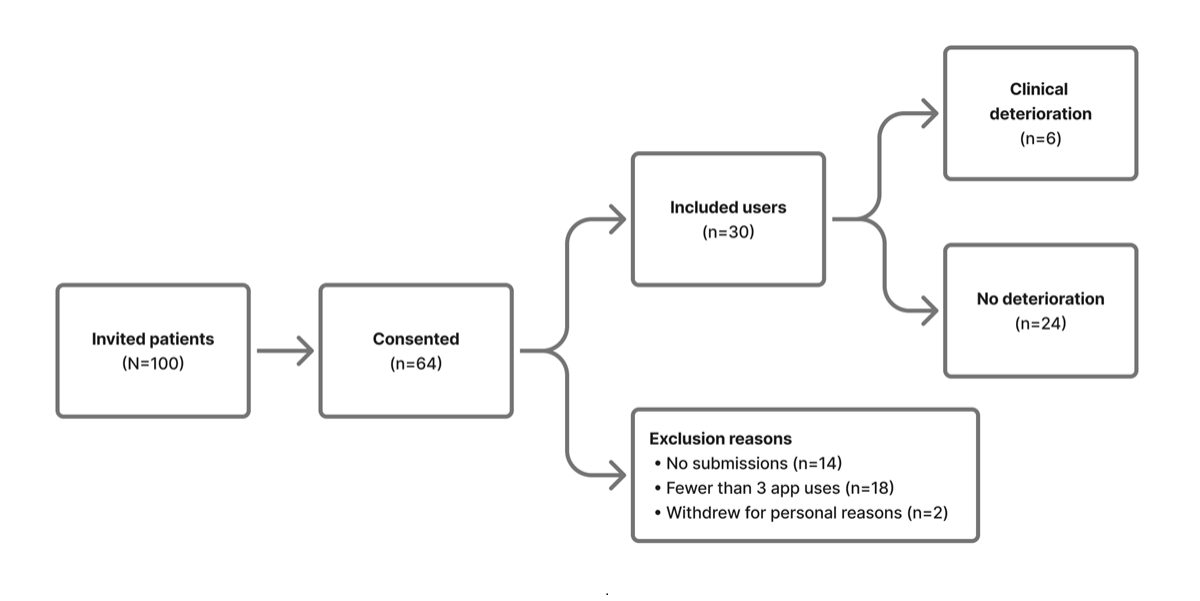
Selection and inclusion of patients.

Of 100 patients approached, 64 (64%) enrolled; after excluding those with <3 total submissions due to access issues (eg, older devices and unstable connections) and 2 midstudy withdrawals, 30 (30%) participants completed the study. Baseline characteristics (enrollment, attrition, sex, and diagnosis) are summarized in Figure 1 and Table 1.

**Table 1:** Baseline characteristics of the patients who used the brain tumor self-monitoring app (N=30).

		Values
Age (years), median (range)		57 (16-78)
**Gender, n (%)**		
	Male	19 (63)
	Female	11 (37)
**Diagnosis category, n (%)**		
	Malignant brain tumor	13 (43)
	Benign brain tumor	11 (37)
	Intracranial hemorrhage	4 (13)
	Primary headache disorder	1 (3)
	Head trauma	1 (3)
**Clinical deterioration, n (%)**		
	Yes	6 (20)
	No	24 (80)

### Outcomes or Definitions

“Clinical deterioration” during follow-up was a composite clinical end point, met when any of the following objective criteria were observed: (1) a decline of ≥20 points in the Karnofsky Performance Status Scale [20,21], (2) new or progressive neurological deficits verified on clinical examination, (3) radiographic progression of intracranial lesions on serial neuroimaging, or (4) clinically significant abnormalities in hematologic or biochemical laboratory results indicating disease progression or systemic decline. Events were ascertained only when patients visited the outpatient clinic or were admitted to the emergency room and were verified by the treating neurosurgeon. To prevent information bias, the treating neurosurgeon adjudicating the clinical deterioration was strictly blinded to the patients’ app-submitted symptom scores. The adjudication relied solely on objective electronic medical records, neuroimaging reports, and standardized clinical examinations obtained during these hospital visits. The “deterioration day” was strictly defined as the date of the patient visit (outpatient or emergency room) during which the objective end point was confirmed and recorded by the physician, rather than a retrospectively estimated date of symptom onset based on patient recall. The remaining participants were classified as having no deterioration over the observation period.

### Intervention: Rule-Based Digital Self-Monitoring Web App

**Figure 2:**
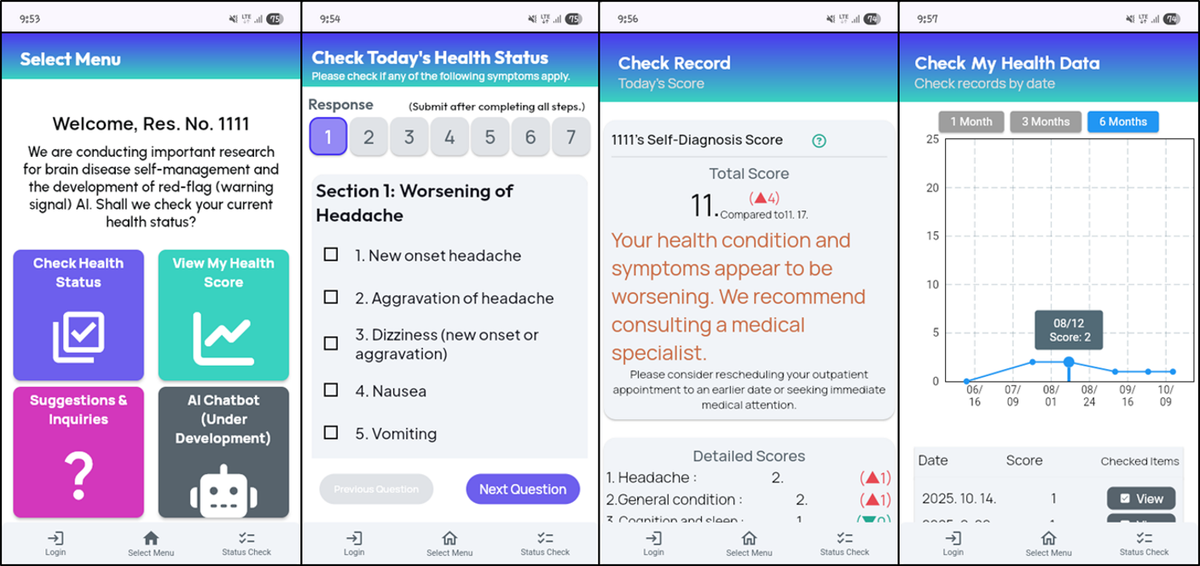
Screenshots of the brain tumor self-monitoring web app (English user interface) function overview. Screen 1 (menu after log-in): 4 tiles—Daily symptom check, View my score trend, Ask my doctor, and artificial intelligence chatbot. Screen 2 (Daily symptom check): 7 symptom categories relevant to neuro-oncology; each category presents 4 to 8 yes or no checkbox items with a step indicator (1-7) and a Next button. Screen 3 (Today’s result): automatically computed daily score with brief interpretation and side-by-side comparison to the most recent prior submission. Screen 4 (My data): time-series plot of cumulative daily scores with tabbed views (graph or table) to review longitudinal trends. The application was originally designed in the Korean language. The figure displays an English-translated version that preserves the same layout and content.

Participants accessed a secure, credentialed web application providing three core functions: (1) *Daily symptom check*—7 sequential pages of brain-disease–related yes or no items (4-10 items per page), with an automatically computed same-day score based on the number of checked items; (2) “My score”—a time-series visualization of daily scores to support self-tracking; and (3) Free-text inquiries—patient-entered concerns routed to the attending neurosurgeon for in-app responses. The overall user interface and task flow are shown in Figure 2.

### Procedures

At discharge, a research coordinator provided onboarding (log-in, tutorial, and practice submission). Ongoing user support was provided through KakaoTalk (Kakao Corp), a widely used mobile instant-messaging app in South Korea [22,23]. Participants were encouraged to complete at least 1 survey per week beginning the day after discharge. One SMS text message reminder was sent weekly (Monday or Tuesday). Outpatient visits proceeded per standard care; no clinical management changes were mandated beyond app use.

### App-Derived Features and Candidate Rules for Deterioration

Each daily submission comprised 51 binary items (checked=1 and unchecked=0). The total score on day *t* was defined as follows:

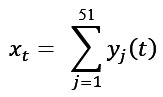

We also computed 7 subcategory subscores 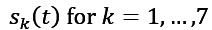 as within-category sums. A clinically curated set of “core” items, C = {6,8,12,13,14,22,23,26,29,30,32,33,34,49}, was prespecified as higher-risk symptoms. The complete list of the 51 symptom items, their category assignments, and whether each item was flagged as “higher risk” is provided in Table S1 in Multimedia Appendix 1.

The higher-risk symptoms (eg, confusion and seizures) were designated a priori as red-flag signs. These symptoms were defined as highly objective indicators of acute neurological deterioration that may pose an immediate threat to patient safety if not evaluated promptly. Although the rule-based alert algorithm is still under development, these red-flag signs were predefined as conditions requiring immediate clinical attention, even in the absence of other symptomatic changes. Clinically, these red-flag signs are operationalized to completely bypass the standard cumulative score rules (R1-R4). If a patient endorses any red-flag item, the system is designed to trigger an immediate on-screen warning instructing the patient to seek urgent medical care (eg, visiting the emergency room), independent of the rule-based thresholds. This red-flag designation is orthogonal to the core set *C* and was used to guide urgent clinical triage.

To reduce within-patient correlation and address class imbalance, the unit of analysis was each patient’s last 3 consecutive submissions (“last-window”: with most recent). For patients with adjudicated clinical deterioration, submissions up to “deterioration day” +1 were retained, and the last window was labeled positive (deterioration=1); otherwise, the window was labeled negative (deterioration=0). Patients with <3 submissions were excluded from rule analysis.

We evaluated 4 simple, interpretable rule families as candidate digital alerts:

- R1: current total score threshold—alert if the current total score is greater than or equal to a fixed threshold T_1_ (ie, *x_i2_* ≥ T_1_)
- R2: net-increase sum threshold (Δ⁺)—alert if the cumulative nonnegative increase in total score over the last 2 intervals meets or exceeds, that is, if 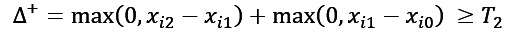
- R3: new-onset combo (core and noncore)—alert if at least C_min_ “core” symptoms and at least N_min_ “noncore” symptoms newly appear between the previous and current windows (ie, items with 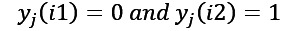).
- R4: number of increasing categories—alert if at least *K* of the 7 category subscores have increased compared with the immediately preceding window (ie, if the count of domains with higher subscore in the current window is ≥ *K* ).

### Threshold Tuning and Model Selection

Thresholds were tuned on observed, discrete values in the last-window dataset using grid search: (1) R1: *T_1_* over empirical integer of *x_i2_*, (2) R2: T_2_ over empirical integer of 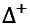, (3) R3**:** (*c_min_, n_min_*)∈{(1,0),(1,1),(1,2),(2,0)}, and (4) R4: K∈{2,3,4}.

**Figure 3:**
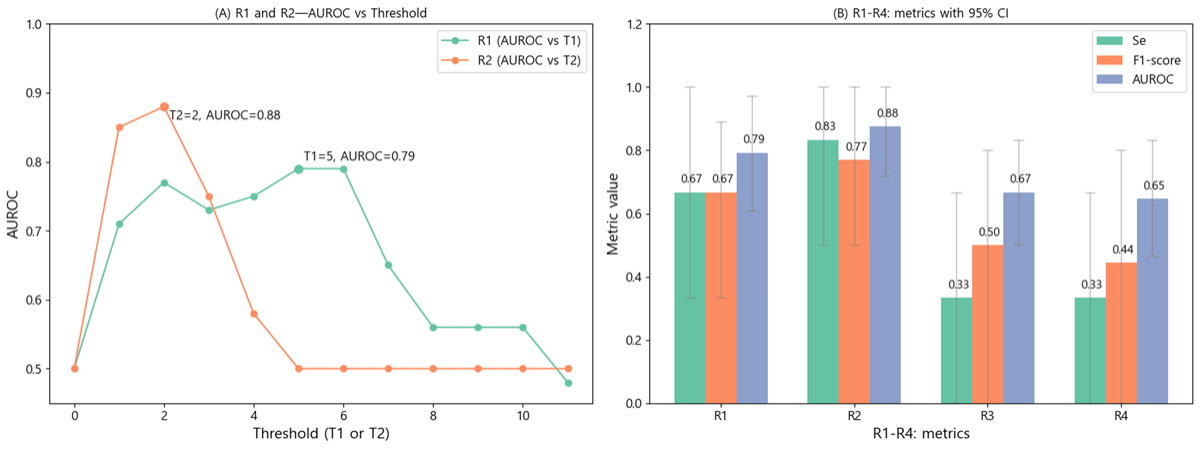
Threshold tuning and rule-wise performance. (A) Area under the receiver operating characteristic (AUROC) vs threshold for R1 (current total score) and R2 (net increase); optima at T₁=5 (AUROC 0.79) and T₂=2 (AUROC 0.88). (B) Comparison of sensitivity (Se), F1-score, and AUROC for R1-R4 at optimized settings, with 95% CIs shown.

The primary selection criterion was area under the receiver operating characteristic (AUROC); ties were broken by *F*_1_-score and then by the positive predictive value (PPV). Final tuned parameters for each rule are reported in Table 2, with performance summarized in Figure 3.

**Table 2:** Candidate rule definitions for digital alerts.

Rule name	Inputs	Calculation	Optimized parameters
R1: current total score threshold	*x*_*i2*_	Positive if *x*_*i2*_ ≥ *T*_1_	*T*_1_=6
R2: net-increase sum threshold	*x*_*i0,*_ *x*_*i1,*_ *x*_*i2*_	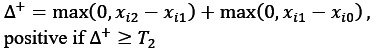	*T*_2_=2
R3: new-onset combo (core and noncore)	*y*_*j*_ *(i1), y*_*j*_ *(i2), C*	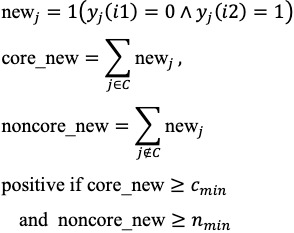	*c*_*min*_=1, *n*_*min*_=1
R4: number of increasing categories	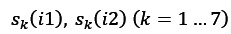	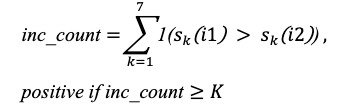	*K*=2

### Statistical Analysis

For each rule, we computed sensitivity, specificity, PPV, negative predictive value, *F*_1_-score, accuracy, AUROC, and area under the precision-recall curve using the binary rule outputs on the last-window sample. Uncertainty was quantified with patient-level bootstrap resampling (5000 replicates) and percentile 95% CIs for each metric. Because our analytic approach restricted the dataset to a single “last-window” observation per patient (ie, analyzing only the final interval before an event or the end of follow-up), each participant contributed exactly 1 independent data point to the evaluation dataset. Consequently, within-patient clustering was inherently avoided in this analysis. Analyses focused on discrimination and precision-recall trade-offs appropriate for rare adverse events in small pilot cohorts. The Python code used for these analyses is available in Multimedia Appendix 2.

To address the missing data resulting from study attrition, we used a complete-case analysis approach for evaluating the alert rules. Because the primary objective of this pilot study was to establish initial technical feasibility and conduct rule-based parameter tuning rather than population-level causal inference, imputation of missing longitudinal submissions was not performed. To assess potential selection bias, baseline demographic and clinical characteristics were compared between the included participants (≥3 submissions) and excluded participants (<3 submissions) using the Mann-Whitney *U* test for continuous variables and chi-square or Fisher exact tests for categorical variables as appropriate. Because the entire last-window cohort was used for both threshold optimization (grid search) and performance evaluation without an independent holdout test set, the reported metrics represent resubstitution (apparent) performance.

### Usability and Satisfaction Instruments

We administered a brief questionnaire, including the System Usability Scale (SUS) and additional items on use frequency and initiation triggers. The survey link was distributed via KakaoTalk; responses were anonymized. A total of 27 responses were received; after excluding 4 respondents who reported never using the app, 23 responses were analyzed. SUS scoring followed standard procedures and is reported alongside satisfaction ratings.

## Results

### App Use and Adherence

Among patients with ≥3 total submissions (analysis cohort; N=30), the mean weekly submission rate was 1.70 surveys per week (range 0.32-6.90); individuals contributed a median of 8.5 (IQR 3-143) submissions over the observation period. The interval between the last 2 submissions (i1→i2) had a median of 17.5 (IQR 15.75; mean 18.4; range 1-105) days, indicating substantial variability and a tendency toward longer gaps near the end of follow-up for some participants. The most recent total score averaged 2.43 points (median 1; range 0-11), and the patient-level mean total score across all submissions averaged 2.21 points (median 1.11; range 0-12.5). Descriptive statistics for use and scores are summarized in Table 3.

A comparison of baseline characteristics between the 30 included participants (completers) and the 34 excluded participants (noncompleters) is presented in Table S2 in Multimedia Appendix 1. There were no statistically significant differences between the 2 groups in terms of age (*P*=.08), sex (*P*=.19), or diagnosis categories (*P*=.36). This lack of significant difference suggests that the initial study attrition was primarily driven by external technological or access issues rather than distinct clinical severity or tumor phenotypes. However, the excluded cohort was significantly older than the included cohort, with a median age of 63.0 years compared to 57.0 years (*P*=.04). There were no significant differences between the two groups in terms of sex (*P*=.19) or diagnosis categories (*P*=.36).

**Table 3:** Descriptive statistics of app use and scores (per patient).

Metric (unit)	Mean (SD)	Min-Max	Median (IQR)
Total submissions per patient (n)	19.03 (30.12)	3-143	8.5 (6-13)
Submission rate (surveys/week)	1.7 (1.7)	0.32-6.9	0.84 (0.53-2.25)
Days between the last 2 submissions: i1→i2 (days)	18.4 (21.25)	1-105	17.5 (4.5-20.0)
Most recent total score: (points)	2.43 (3.06)	0-11	1 (0.0-3.25)
Patient-level mean total score across submissions	2.21 (2.76)	0-12.5	1.11 (0.29-3.47)

### Feature Discovery and Threshold Optimization

We conducted grid searches to tune thresholds for the 4 prespecified rule families (R1-R4) defined in the Methods section. The optimized parameters are reported in Table 2. For visualization, AUROC was plotted against the candidate thresholds for R1 (current total score, *T*_1_) and R2 (net increase, *T*_2_); both curves showed clear internal maxima, with peak performance at *T*_1_=5 and *T*_1_=2, respectively (Figure 3A). These findings support the face-valid expectation that (1) a moderately elevated current score and (2) short-horizon net worsening each contribute usefully to discrimination.

### Rule-Wise Performance Comparison

At the optimized settings, R2 (net increase) showed the best overall balance (AUROC 0.875; sensitivity 0.833; specificity 0.917; PPV 0.714; negative predictive value 0.957; *F*_1_-score 0.769; accuracy 0.900), outperforming other rules on discrimination and *F*_1_-score while preserving high specificity (Figure 3B; Table 4). R1 performed second best (AUROC 0.792; *F*_1_-score 0.667; accuracy 0.867), while R3 and R4 traded sensitivity for specificity (R3: specificity 1.000; sensitivity 0.333; AUROC 0.667 and R4: specificity 0.958; sensitivity 0.333; AUROC 0.646), indicating more conservative alerting with reduced case finding. Bootstrap 95% CIs for sensitivity, *F*_1_-score, and AUROC are shown as error bars in Figure 3B. It is important to note that due to the small sample size and the low number of clinical deterioration events (n=6), these CIs are wide. This indicates that the point estimates (eg, the AUROC of 0.88 for R2) are statistically unstable and should be interpreted with caution. Nevertheless, the point estimates for R2 remain consistently highest across metrics, supporting the value of short-horizon worsening (change from the immediately prior submission) as a digital signal of deterioration.

**Table 4:** Performance of candidate alert rules (R1-R4) on the last-window dataset at optimized thresholds.

Rule^a^	Sensitivity	Specificity	PPV^b^	NPV^c^	*F*_1_-score	Accuracy	AUROC^d^
R1	0.667 (0.33-1.00)	0.917 (0.85-1.00)	0.667 (0.33-1.00)	0.917 (0.85-1.00)	0.667 (0.33-0.90)	0.867 (0.78-0.95)	0.792 (0.61-0.97)
R2	0.833 (0.50-1.00)	0.917 (0.85-1.00)	0.714 (0.33-1.00)	0.957 (0.92-1.00)	0.769 (0.50-1.00)	0.900 (0.83-1.00)	0.875 (0.72-1.00)
R3	0.333 (0.00-0.67)	1.000 (1.00-1.00)	1.000 (1.00-1.00)	0.857 (0.77-0.95)	0.500 (0.00-0.80)	0.867 (0.78-0.95)	0.667 (0.50-0.83)
R4	0.333 (0.00-0.67)	0.958 (0.92-1.00)	0.667 (0.00-1.00)	0.852 (0.75-0.95)	0.444 (0.00-0.80)	0.833 (0.74-0.94)	0.646 (0.47-0.83)

^a^Data are presented as point estimates with 95% bootstrap CIs in parentheses.

^b^PPV: positive predictive value.

^c^NPV: negative predictive value.

^d^AUROC: area under the receiver operating characteristic.

### Item-Level Response Differences

**Figure 4:**
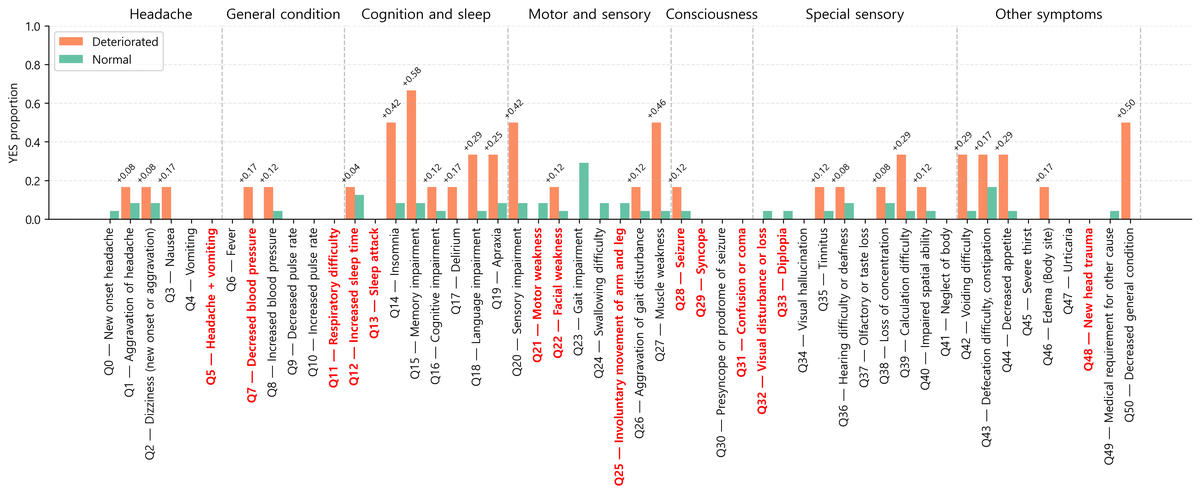
Item-level yes-response proportions by symptom category (7 domains) comparing deteriorated (orange) vs normal (green) windows. Dashed lines mark category boundaries, and red labels denote prespecified core items. Numbers above bars denote the between-group proportion difference (deteriorated−normal). Larger between-group differences are notable in the sleep and cognition and other symptoms domains.

Item-wise yes-response rates were contrasted between patients with adjudicated deterioration and those without (Figure 4). When grouped by the 7 symptom categories, the “cognition and sleep” and “other symptoms” domains showed notably higher affirmative rates in the deterioration group. At the item level, “memory impairment,” “decreased general condition,” and “muscle weakness” ranked among the largest between-group differences. Conversely, several items predesignated as “core” due to presumed clinical severity were infrequently endorsed in both groups, limiting their utility as direct triggers in this dataset. Taken together, these patterns indicate that comparatively *softer* but commonly reported symptoms may carry earlier signal for deterioration than rarer, high-severity items.

### Usability (SUS)

**Figure 5:**
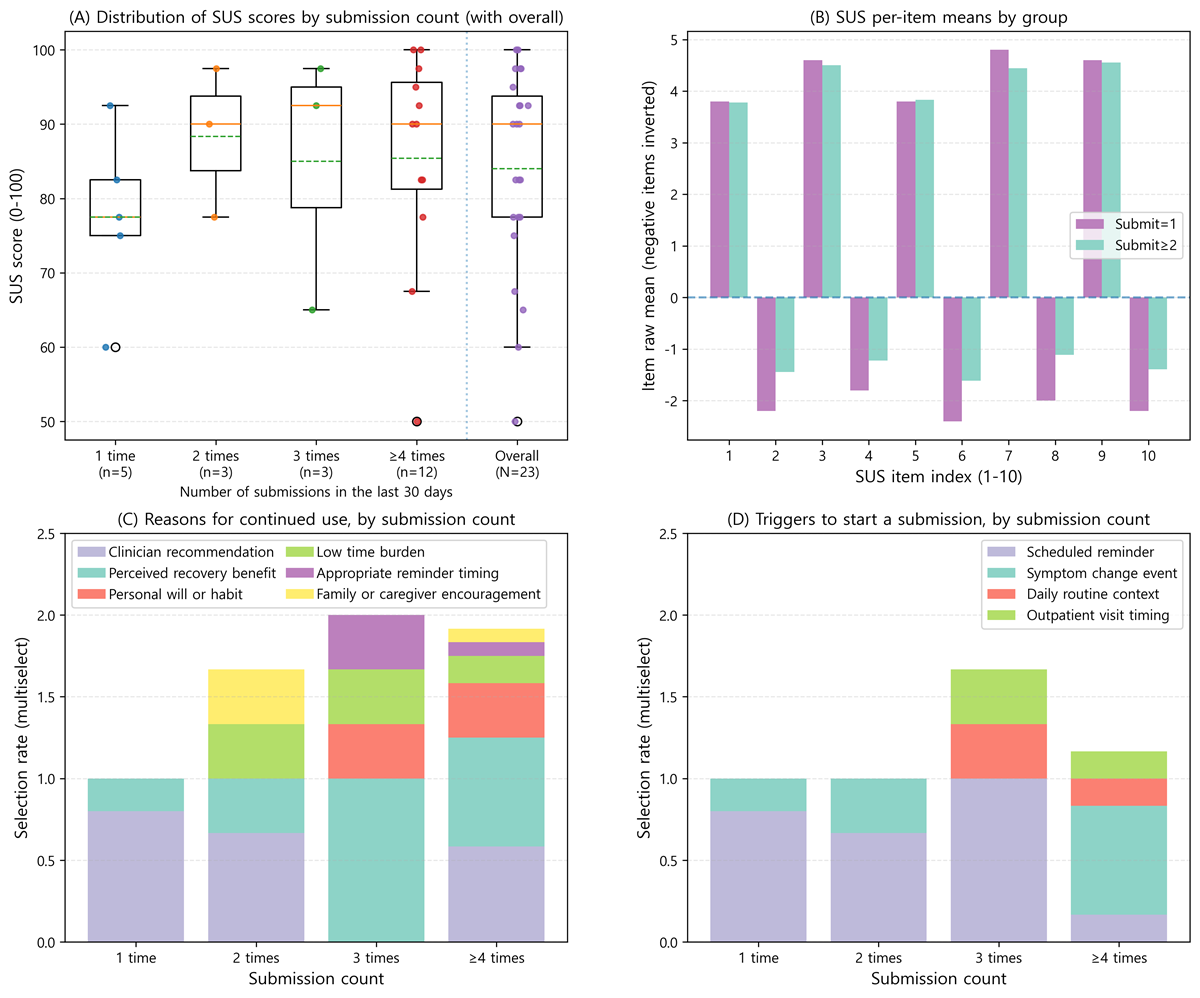
Usability and adherence-related survey results. (A) System Usability Scale (SUS) total score distributions (0-100) by submission-count group and overall (boxplots with individual points; N=23 app users). (B) Mean SUS item scores by group (1 submission vs ≥2 submissions); reverse-scored items appear below 0 after centering. (C) Reasons for continued use (multiresponse) by submission-count group—stacked proportions. (D) Triggers to start a submission (multiresponse) by submission-count group—stacked proportions.

Of 27 questionnaire respondents, 23 (85%) reported at least 1 instance of app use and were included in the SUS analysis. The SUS total score distribution is shown in Figure 5A (mean 84.0, median 90.0), indicating generally favorable perceived usability. Participants with only 1 submission exhibited a lower SUS distribution than those with ≥2 submissions, consistent with lower adherence among users reporting poorer usability. To probe domain-level patterns, item means were compared between the 1-submission and ≥2-submission groups (Figure 5B): differences were modest for standard (odd-numbered) items, while reverse-scored (even-numbered) items were more negative in the single-submission group. This indicates that infrequent users tend to perceive higher complexity or lower consistency in relation to app use.

### Adherence-Related Survey Findings

Multiresponse questions on (1) reasons for continued use and (2) triggers to initiate a submission are summarized in Figures 5C and 5D. Participants with higher submission counts more frequently selected “personal will or habit” and “perceived recovery benefit” as reasons for sustained use. For initiation triggers, higher-adherence users more often endorsed cues embedded in daily routines. Together, these findings imply that emphasizing perceived clinical usefulness and integrating reminders into routine time points (eg, after lunch) may enhance adherence in future iterations of the intervention.

## Discussion

### Principal Findings

This preliminary study demonstrated the feasibility and patient acceptability of a rule-based smartphone and web application for self-monitoring in individuals recovering from brain tumor surgery. Following major neurosurgical procedures, even minor physical or neurological changes can provoke significant anxiety [24]. By providing a structured, medically validated alternative to unverified online information, this platform supports objective symptom tracking and bridges the gap between intermittent in-person clinic visits [25-29]. The high usability scores (mean SUS 84.0) suggest that patients are willing to engage with digital health tools during the vulnerable postoperative period, a willingness likely bolstered by the personalized oversight of their treating neurosurgeon. Furthermore, our evaluation of simple heuristic rules, particularly the short-horizon worsening rule (R2), demonstrated preliminary discriminative potential for capturing early signals of clinical deterioration.

### Clinical Implications and Workflow Integration

While the technical performance of these heuristic rules demonstrates initial feasibility, excellent technical accuracy alone is insufficient for a viable clinical decision support system. A critical challenge for real-world implementation is the risk of alert fatigue. Generating an excessive number of false alarms can easily overwhelm clinical staff and disrupt workflows. Therefore, future deployments of this system will use a structured, tiered triage protocol. Rather than directly interrupting the attending neurosurgeon, a breached cumulative threshold could trigger an automated recommendation for the patient to reschedule a clinic visit or route the alert to a specialized triage nurse for preliminary review. Conversely, prespecified “red-flag” symptoms (eg, seizures) are operationalized as distinct medical emergencies; endorsing these items bypasses the cumulative score rules entirely to provide instantaneous safety instructions, ensuring critically urgent cases receive immediate attention.

From a technical standpoint, the current platform evaluates simple, prespecified threshold rules, which serve merely as a foundational step. The long-term vision of this project is to establish an advanced artificial intelligence–driven early warning framework [8,30,31]. By aggregating longitudinal patient-reported symptom trajectories and eventually integrating continuous physiological data from wearable devices [32-35], future machine learning algorithms could autonomously identify subtle, multivariate deviations. This transition represents a paradigm shift from a passive, provider-centered framework to an active, patient-engaged model of postoperative neurosurgical care [36-40].

### Limitations

As with all pilot studies, several limitations should be acknowledged. First, the small sample size (N=30) and the low number of clinical deterioration events (n=6) mean that the discriminative performance metrics—including the high AUROC observed for R2—are statistically unstable and exhibit wide CIs. These results must be interpreted strictly as preliminary feasibility signals rather than definitive validations. Additionally, to accommodate data sparsity during this pilot phase, we used a simplified “last-window” heuristic and a complete-case analysis. This approach does not fully leverage the longitudinal nature of the data or account for within-patient correlation. Future investigations must address these analytical limitations by using advanced longitudinal methodologies, such as mixed-effects models or time-series machine learning algorithms, in larger independent cohorts. Furthermore, defining the clinical event by the actual hospital visit date introduces potential lead-time bias. Because patients may delay seeking care despite experiencing worsening symptoms, the observed interval between an app threshold breach and the clinical event may overestimate the system’s true early warning capability. Additionally, our threshold parameters were optimized and evaluated on the exact same dataset. Consequently, our findings represent apparent (or resubstitution) performance that is inherently subject to optimistic bias. These rule thresholds must be rigorously evaluated and calibrated on independent, external test sets in future larger-scale studies.

Second, the substantial attrition rate (34/64, 53%) introduces potential selection bias. Although baseline clinical characteristics did not differ significantly between completers and noncompleters, the dropout group tended to be older (median 63.0 vs 57.0 years; *P*=.04). The platform currently targets patients with mild symptoms capable of independent daily activities, and the high usability scores likely reflect a technologically adept subgroup. However, patients with brain tumors frequently experience postoperative cognitive deficits, visual impairments, or motor limitations that hinder standard smartphone interaction. To address these significant digital literacy and physical barriers, future iterations will incorporate tailored adaptations, including caregiver proxy-reporting functionalities and natural conversational (chatbot) interfaces currently under development. Advanced statistical methods such as multiple imputation should also be used to handle missing data robustly.

Finally, this study was conducted at a single tertiary medical center in South Korea, an environment with exceptionally high smartphone penetration, reliable digital infrastructure, and specific health care follow-up protocols. Consequently, the high engagement rates and feasibility observed may not directly translate to other global health care systems or populations with different socioeconomic constraints. Multicenter validations across diverse geographic contexts are required to externally validate these digital self-monitoring tools.

### Conclusions

Our preliminary report provides early evidence that smartphone-based self-monitoring systems are both feasible and acceptable among patients with brain tumors. Beyond usability, this research lays the groundwork for the next phase of development—building predictive, artificial intelligence–powered early warning algorithms that can autonomously identify high-risk symptom patterns and deliver timely alerts. Such an approach has the potential to redefine postoperative neurosurgical care, enhance digital health care accessibility, and ultimately improve patient safety and long-term outcomes.

## Appendix

Multimedia Appendix 1 Daily self-monitoring questionnaire items and baseline patient characteristics.

Multimedia Appendix 2 Python source code for statistical analysis, bootstrap CI estimation, rule-based alert evaluation, and figure generation.

## Abbreviations

AUROC: area under the receiver operating characteristic
PPV: positive predictive value
SUS: System Usability Scale

## Data Availability

The datasets generated or analyzed during this study are available from the corresponding author on reasonable request.
