# Supplementary material for: Preliminary Usability Assessment of a Rule-Based Digital Self-Monitoring Platform for Patients With Brain Tumors Toward Digital Early Warning Systems: Pilot Feasibility Study
Source: JMIR Form Res. 2026 Apr 17;10:e87928. doi: 10.2196/87928 (PMC13135161; doi:10.2196/87928)
Supplement: Multimedia Appendix 1 [file formative_v10i1e87928_app1.docx]

# Multimedia Appendix 1

# Feasibility and Preliminary Usability Assessment of a Rule-Based Digital Self-Monitoring Platform for Patients With Brain Tumors: A Pilot Study Toward Digital Early Warning Systems

Table S1. List of 51 binary symptom items included in the daily self-monitoring questionnaire, grouped by symptom category, with designation of high-risk (“red-flag”) items (High Risk: 1 = yes, 0 = no).

| **Index** | **Category** | **Name of Symptoms** | **High Risk**  (1=Yes,  0=No) |
| --- | --- | --- | --- |
| 1 | Headache | New onset headache | 0 |
| 2 |  | Aggravation of headache | 0 |
| 3 |  | Dizziness (new onset or aggravation) | 0 |
| 4 |  | Nausea | 0 |
| 5 |  | Vomiting | 0 |
| 6 |  | Headache + vomiting | 1 |
| 7 | General condition | Fever | 0 |
| 8 |  | **Decresed blood pressure** | **1** |
| 9 |  | Increased blood pressure | 0 |
| 10 |  | Decreased pulse rate | 0 |
| 11 |  | Increased pulse rate | 0 |
| 12 |  | **Respiratory difficulty** | **1** |
| 13 | Cognition and sleep | **Increased sleep time** | **1** |
| 14 |  | **Sleep attack** | **1** |
| 15 |  | Insomnia | 0 |
| 16 |  | Memory impairment | 0 |
| 17 |  | Cognitive impairment | 0 |
| 18 |  | Delirium | 0 |
| 19 |  | Language impairment | 0 |
| 20 |  | Apraxia | 0 |
| 21 | Motor and sensory | Sensory impairment | 0 |
| 22 |  | **Motor weakness** | **1** |
| 23 |  | **Facial weakness** | **1** |
| 24 |  | Gait impairment | 0 |
| 25 |  | Swallowing difficulty | 0 |
| 26 |  | **Involuntary movement of arm and leg** | **1** |
| 27 |  | Aggravation of gait disturbance | 0 |
| 28 |  | Muscle weakness | 0 |
| 29 | Consciousness | **Seizure** | **1** |
| 30 |  | **Syncope** | **1** |
| 31 |  | Presyncope or prodrome of seizure | 0 |
| 32 |  | **Confusion or coma** | **1** |
| 33 | Special sensory | **Visual disturbance or loss** | **1** |
| 34 |  | **Diplopia** | **1** |
| 35 |  | Visual hallucination | 0 |
| 36 |  | Tinnitus | 0 |
| 37 |  | Hearing difficulty or deafness | 0 |
| 38 |  | Olfactory or taste loss | 0 |
| 39 |  | Loss of concentration | 0 |
| 40 |  | Calculation difficulty | 0 |
| 41 |  | Impaired spatial ability | 0 |
| 42 |  | Neglect of body | 0 |
| 43 | Other symptoms | Voiding difficulty | 0 |
| 44 |  | Defecation difficulty, constipation | 0 |
| 45 |  | Decreased appetite | 0 |
| 46 |  | Severe thirst | 0 |
| 47 |  | Edema (Body site) | 0 |
| 48 |  | Urticaria | 0 |
| 49 |  | **New head trauma** | **1** |
| 50 |  | Medical requirement for other cause | 0 |
| 51 |  | Decreased general condition | 0 |

Table S2. Comparison of baseline characteristics between excluded (non-completers) and included (completers) patients.

| **Characteristics** | **Excluded (n=34)** | **Included (n=30)** | **p-value** |
| --- | --- | --- | --- |
| Age, median (IQR) | 63.0 (51.0–71.8) | 57.0 (47.2–64.5) | 0.080* |
| **Sex, n (%)** |  |  | 0.198† |
| - Female | 15 (44.1%) | 19 (63.3%) |  |
| - Male | 19 (55.9%) | 11 (36.7%) |  |
| **Diagnosis category, n (%)** |  |  | 0.364† |
| - Malignant brain tumor | 14 (41.2%) | 13 (43.3%) |  |
| - Benign brain tumor | 15 (44.1%) | 11 (36.7%) |  |
| - Intracranial hemorrhage | 1 (2.9%) | 4 (13.3%) |  |
| - Head trauma | 3 (8.8%) | 1 (3.3%) |  |
| - Primary headache disorder | 1 (2.9%) | 1 (3.3%) |  |

*Calculated using the Mann-Whitney U test.

†Calculated using the Chi-square or Fisher’s exact test. (Note: Total diagnoses for excluded patients in subcategories reflect available clinical data).
